# Islet-Like Clusters Derived from Mesenchymal Stem Cells in Wharton's Jelly of the Human Umbilical Cord for Transplantation to Control Type 1 Diabetes

**DOI:** 10.1371/journal.pone.0001451

**Published:** 2008-01-16

**Authors:** Kuo Ching Chao, Kuo Fang Chao, Yu Show Fu, Shing Hwa Liu

**Affiliations:** 1 Institute of Toxicology, College of Medicine, National Taiwan University, Taipei, Taiwan; 2 Department of Anatomy, School of Medicine, National Yang-Ming University, Taipei, Taiwan; 3 Department of Surgery, National Taiwan University Hospital, Taipei, Taiwan; University of California at Los Angeles, United States of America

## Abstract

**Background:**

There is a widespread interest in developing renewable sources of islet-replacement tissue for type I diabetes mellitus. Human mesenchymal cells isolated from the Wharton's jelly of the umbilical cord (HUMSCs), which can be easily obtained and processed compared with embryonic and bone marrow stem cells, possess stem cell properties. HUMSCs may be a valuable source for the generation of islets.

**Methodology and Principal Findings:**

HUMSCs were induced to transform into islet-like cell clusters *in vitro* through stepwise culturing in neuron-conditioned medium. To assess the functional stability of the islet-like cell clusters *in vivo*, these cell clusters were transplanted into the liver of streptozotocin-induced diabetic rats via laparotomy. Glucose tolerance was measured on week 12 after transplantation accompanied with immunohistochemistry and electron microscopy analysis. These islet-like cell clusters were shown to contain human C-peptide and release human insulin in response to physiological glucose levels. Real-time RT-PCR detected the expressions of insulin and other pancreatic β-cell-related genes (*Pdx1*, *Hlxb9*, *Nkx2.2*, *Nkx6.1*, and *Glut-2*) in these islet-like cell clusters. The hyperglycemia and glucose intolerance in streptozotocin-induced diabetic rats was significantly alleviated after xenotransplantation of islet-like cell clusters, without the use of immunosuppressants. In addition to the existence of islet-like cell clusters in the liver, some special fused liver cells were also found, which characterized by human insulin and nuclei-positive staining and possessing secretory granules.

**Conclusions and Significance:**

In this study, we successfully differentiate HUMSCs into mature islet-like cell clusters, and these islet-like cell clusters possess insulin-producing ability *in vitro* and *in vivo*. HUMSCs in Wharton's Jelly of the umbilical cord seem to be the preferential source of stem cells to convert into insulin-producing cells, because of the large potential donor pool, its rapid availability, no risk of discomfort for the donor, and low risk of rejection.

## Introduction

Type 1 diabetes accounts for only about 5–10% of all cases of diabetes; however, its incidence continues to increase worldwide and it has serious short-term and long-term implications [1]. Transplantation of pancreatic islet cells as a potential cure for type I diabetes mellitus has become the subject of intense interest over the past two decades [2]–[4]. A protocol, characterized by the infusion of multiple, fresh donor islets followed by the host immune suppression with nonsteroidal immunosuppressive regimen, has proved to be effective in treating severe cases of type I diabetes [2]. However, the problem of the worldwide shortage of transplant-ready islets has yet to be resolved. Moreover, islet transplantation has been hampered by immune rejection and recurrent attacks against islets by the underlying autoimmunity [2], [5]. Immunosuppressive regimens are capable of preventing islet failure for months to years, but these regimens may increase the risk for specific malignancies and opportunistic infections [6]. Ironically, all commonly used immunosuppressive drugs (steroids, calcineurin inhibitors, and rapamycin) have been reported to have adverse effects on pancreatic β-cells [7]. Therefore, these factors motivate efforts to develop renewable sources of islet-replacement tissue.

Many studies have made efforts to expand pancreatic islets and to develop renewable sources of islet-replacement tissue. Recent studies have shown that embryonic stem cells [8], [9], pancreatic ductal cells [10], [11], hepatic stem cells [12], neural progenitor cells [13], bone marrow-derived cells [14], [15], or umbilical cord blood cells [16], [17], may be possible sources to derive transplantable insulin-producing cells or islet-replacement tissues. In most of these studies, with respect to the test of functional β-cell generation, the insulin-producing cells or tissues were transplant into immunocompromised recipient animal with or without streptozotocin-induced diabetes mellitus. Both embryonic stem cells and fetus-derived stem cells have ethical problems that impede their application into the clinic. Postnatal stem cells offer fewer concerns in terms of moral issues.

Human mesenchymal cells isolated from the Wharton's jelly of the umbilical cord, which can be easily obtained and processed compared to embryonic and bone marrow stem cells, possess stem cell properties [18]–[20]. These postnatal stem cells have broad developmental potential, which could be induced to differentiate into neuron-like cells, osteogenic, chondrogenic, adipogenic, and myogenic cells in vitro, without ethical problems [18]–[20]. Recent studies showed that no rejections occur even after xenotransplantation of post-differentiated umbilical cord mesenchymal stem cells without immunosuppression therapy [21], [22]. The potential of umbilical cord mesenchymal stem cells to form insulin-producing cells has not yet been evaluated. Therefore, the availability of human umbilical cord mesenchymal stem cells allowed us to investigate whether these stem cells could develop into glucose-responsive insulin-producing cells. Here, we describe experimental strategy for developing pancreatic islet-like cell clusters directly from human mesenchymal stem cells isolated from Wharton's jelly of the umbilical cord.

## Results

### Four stage differentiation protocol and morphological changes of HUMSCs

**Figure 1:**
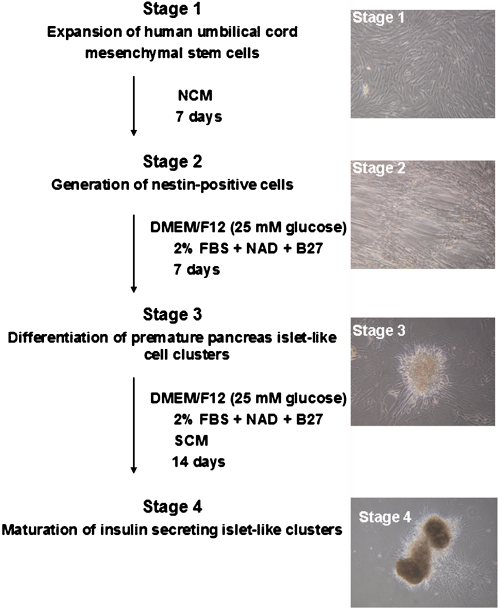
Outline of differentiation protocol and stage-specific cell cluster morphology. Essential factor manipulations at each stage were also shown. Original magnification: ×40.

**Figure 2:**
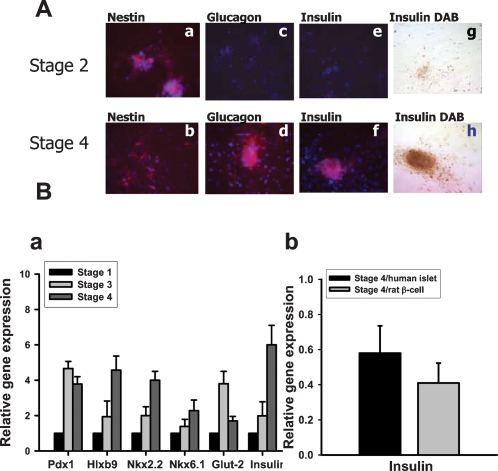
Detections of nestin, glucagons, insulin, and pancreatic β-cell-related genes in stage-specific cell cluster. In A, immunocytochemical detection of nestin, glucagon and insulin in stage 2 and stage 4 of differentiated human umbilical cord mesenchymal stem cells. Immunofluorescent images were obtained by confocal microscopy and are representative of at least 6 samples for each antibody. In g and h, DAB was a substrate for the immunostaining of insulin. Results shown are representative of three independent experiments. Original magnification: ×40. In B-a, real-time RT-PCR detected the mRNA expressions of insulin and other pancreatic β-cell-related genes, such as *Pdx-1*, *Hlxb9*, *Nkx2.2*, *Nkx6.1*, and *Glut-2* in these islet-like cell clusters. In B-b, real-time RT-PCR detected the insulin mRNA expressions in stage 4 cell clusters comparing to human islets or rat β-cell line RIN-m5F. Data are presented as means±SEM from three independent experiments.

The neuronal conditioned medium (NCM) was made from rat brain, on which HUMSCs were growing and differentiated into nestin positive cells. The level of nestin-positive cells reached a peak on the day 7, represented by red fluorescence detected on immunocytochemistry study using anti-nesting antibodies (Figs. 1 and 2). Under inversed microscope, undifferentiated HUMSCs (stage 1) were typically adherent spindle shape (Fig. 1). These spindle-like cells became aggregates (stage 2), and some formed clusters under neuronal conditioned medium (NCM) (Figs. 1 and 2). These spindle-shape cells became round or oval-type cells in the medium containing high glucose (25 mM), insulin, nicotinamide, and B27, and later formed clusters (stage 3; Fig. 1). These cells continued to differentiate and eventually resulted in larger clusters under stage 3 medium supplemented with SCM (stage 4; Figs. 1 and 2).

Immunocytochemistry was performed to test nestin, insulin, and glucogan protein expressions in stage 2 and stage 4 of HUMSCs differentiation. Nestin was regarded as an important marker for initiation of islet cells differentiation. There were no insulin and glucagon expressions in cells of stage 2 of HUMSCs differentiation, but it showed more nestin-staining cells than cells in stage 4. In cells of stage 4, there were more insulin and glucagon expression (Fig. 2A). Moreover, by using real-time reverse transcription-polymerase chain reaction, the mRNA expressions of insulin and other pancreatic β-cell-related genes, such as *Pdx1*, *Hlxb9*, *Nkx2.2*, *Nkx6.1*, and *Glut-2* were observed to be significantly activated in stage 4 islet-like cell clusters (Fig. 2B-a), but it was not observed in stage 2 cells (data not shown). The comparison of insulin mRNA expression levels in stage 4 islet-like cell clusters relative to isolated human islets as well as a rat β-cell line RIN-m5F was also tested. As shown in figure 2B-b, the relative insulin mRNA expressions in stage 4 islet-like clusters/human islets and stage 4 islet-like clusters/RIN-m5F were 0.58±0.16 and 0.41±0.12, respectively.

**Figure 3:**
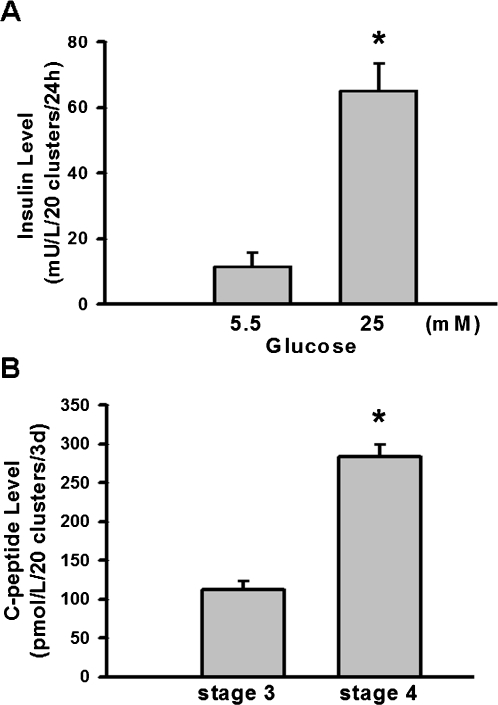
In vitro insulin and C-peptide secretion assay of islet-like cell clusters. In A, insulin release in response to physiological (5.5 mM) and high (25 mM) glucose concentrations of stage 4 cell cluster was measured after 24 h incubation. In B, C-peptide secretion of stage 3 and stage 4 cell clusters was detected. All data are presented as means±SEM (n = 6 in A; n = 8 in B).

The cell clusters in stage 4 secreted lower levels of insulin on low glucose (5.5 mM) DMEM medium, but secreted higher levels of insulin on high glucose (25 mM) DMEM medium, which was similar to pancreatic β-cells (Fig. 3A). Moreover, C-peptide, a product of insulin formation, represents new insulin formation instead of insulin absorption from the medium. The cell clusters in stage 4 secreted much higher levels of C-peptide than the cell clusters in stage 3 did (Fig. 3B). These results show that stage 4 cell clusters release insulin in response to the physiological glucose concentrations *in vitro*.

### Improved glucose regulation and circulating insulin levels in STZ-induced diabetic rats by stage 4 cell clusters transplantation

**Figure 4:**
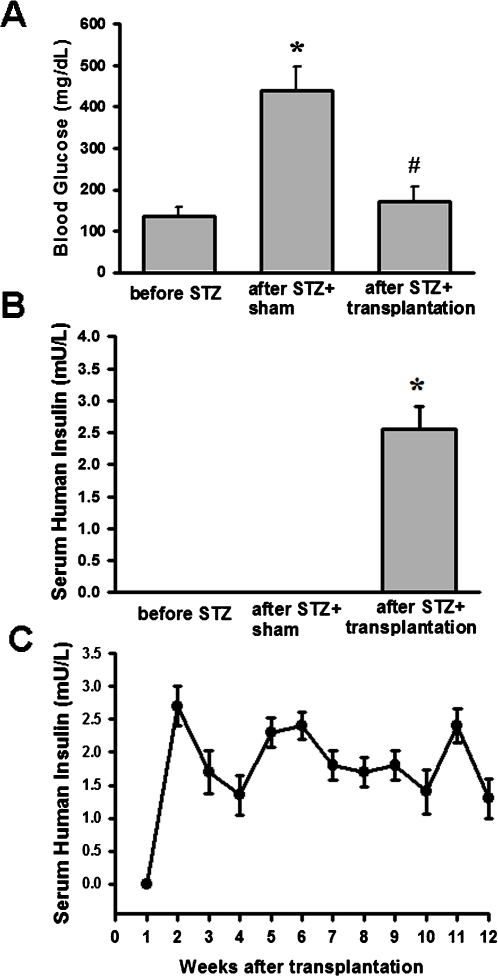
Changes of blood glucose and serum human insulin levels in STZ-diabetic rats after clusters transplantation. Blood glucose (A) and serum human insulin (B) detection 1-week after sham or stage 4 islet-like cell cluster transplantation in STZ-induced diabetic rats. Random-fed blood glucose levels were measured in all groups. In C, time-course changes of serum human insulin levels after transplantation. All data are presented as means±SEM (n = 6 in each group). * P<0.05 as compared with before STZ. ^#^ P<0.05 as compared with after STZ.

To assess the functional stability of the islet-like cell clusters *in vivo*, islet-like cell clusters were transplanted into the liver of STZ-induced diabetic rats via laparotomy. One week after sham transplantation, STZ-treated control rats became hyperglycemia (439.33±58.41 mg/dl; Fig. 4A). In STZ-induced diabetic rats xenografted with stage 4 islet-like cell clusters, hyperglycemia was markedly attenuated (171.17±36.90 mg/dl; Fig. 4A) compared with sham-transplanted controls during random-feeding. There is no immunosuppressant used in these islet-like cell clusters-transplanted rats. Moreover, serum human insulin level was also detected during random-feeding. The serum human insulin level was near absent in sham-transplanted control and STZ-treated rats. The Insulin levels in STZ-treated rats transplanted with stage 4 islet-like cell clusters for one week were increased (2.56±0.34 mU/L; Fig. 4B) and maintained to week 12 after transplantation (Fig. 4C). The cross-reaction between human insulin and rat insulin is very low (about 0.7%) in insulin ELISA kit (Mercodia, Sweden).

**Figure 5:**
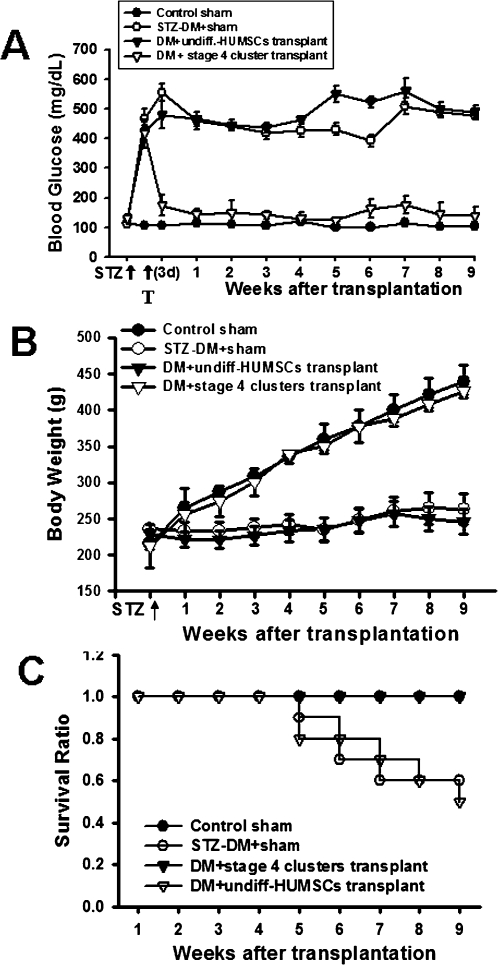
Functional transplantation of stage 4 islet-like cell clusters alleviates hyperglycemia, decreased body weight and decreased survival ratio in STZ-induced diabetic rats. STZ-induced diabetic rats (blood glucose levels >400 mg/dl) were transplanted with stage 4 insulin secreting islet-like clusters. Random-fed blood glucose levels were measured in untreated control rats with sham, STZ-treated rats with sham, and STZ-treated rats with transplantation of islet-like cell clusters or undifferentiated HUMSCs (undiff-HUMSCs). The changes in blood glucose (A), body weight (B) and survival ratio (C) were observed. All data are presented as means±SEM (n = 6 in each group).

**Figure 6:**
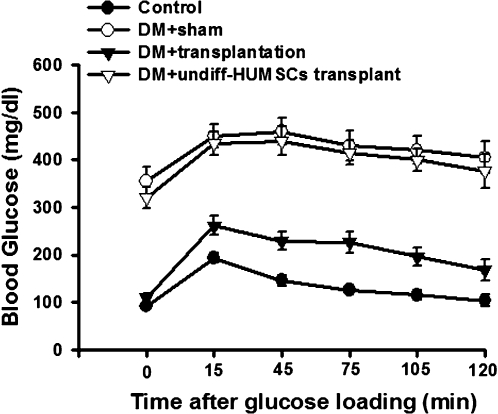
Correction of glucose intolerance in STZ-induced diabetic rats transplanted with stage 4 islet-like cell clusters. Intraperitoneal glucose tolerance test performed on post-transplantation week 12 in untreated control rats with sham, STZ-treated rats with sham, and STZ-treated rats with transplantation of islet-like cell clusters or undifferentiated HUMSCs (undiff-HUMSCs). All data are presented as means±SEM (n = 6 in each group).

**Figure 7:**
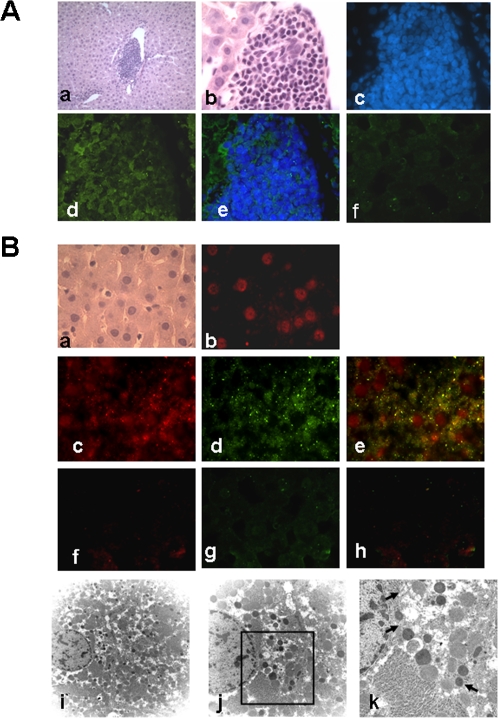
Histological analysis of the liver of STZ-induced diabetic rats transplanted with stage 4 islet-like cell clusters for 12 weeks. In A-a and -b, hematoxylin and eosin-stained sections of liver with cell cluster. In A-c-e, individual (c, d) and merged (e) fluorescent images of liver sections from STZ-induced diabetic rats transplanted with islet-like cell clusters. Sections were stained with a anti-human insulin antibody (green) (d and e) with or without 4,6-diamidino-2-phenylindole (DAPI; blue) (c and e). Image b to e is from consecutive section of a. Original magnification: ×20 (a); ×100 (b-e). In A-f, individual fluorescent image of liver section from STZ-induced diabetic rats (sham, without transplantation of islet-like cell clusters). Section was stained with an anti-human insulin antibody. In B-a, hematoxylin and eosin-stained sections of liver. In B-b-e, individual (b, c and d) and merged (e) fluorescent images of liver sections from STZ-induced diabetic rats transplanted with islet-like cell clusters. Sections were stained with an anti-human nuclei antibody (red) (b, c and e) without or with anti-human insulin antibody (green) (d and e). The human origin of the engrafted cells was established by immunohistochemistry by using the anti-human nuclei monoclonal antibody. Image c to e is from consecutive section of a. Original magnification: ×100. In B-f-h, individual (f and g) and merged (h) fluorescent images of liver sections from STZ-induced diabetic rats (sham, without transplantation of islet-like cell clusters). Sections were stained with an anti-human nuclei antibody (f and h) without or with anti-human insulin antibody (g). In B-i-k, electron microscopy of secretory granules within liver cells of liver sections from STZ-induced diabetic rats transplanted with islet-like cell clusters. The presence of secretory granules with crystalline formation is evident at high magnification (arrow). Original magnification: ×5000 (i); ×8000 (j); ×20000 (k). All results shown are representative of three independent experiments.

The level of hyperglycemia was lowered starting 3 days after transplantation. Hyperglycemia was normalized thereafter and stable blood glucose levels were maintained for >9 weeks, until the experiment was terminated for immunohistochemical analysis (Fig. 5A). No hypoglycemia was noted at the end of the experiment. The STZ-diabetic rats lost weight persistently and decreased the survival ratio, which were also reversed after transplantation (Figs. 5B and C). To further evaluate the function of the implanted islet-like cell clusters, we performed an IPGT test on normal, STZ-diabetic and transplanted STZ-diabetic rats after 12-week transplantation. As shown in Figure 6, the result of IPGT testing indicated a normal rate of glucose clearance after transplantation of islet-like cell clusters, but not the transplantation of undifferentiated HUMSCs. Subsequently, immunohistochemical analysis detected human insulin immunofluorescence staining in the transplanted islet-like cell clusters in the liver (Fig. 7A).

Unexpectedly, human insulin immunofluorescence staining was also detected in the non-cell clusters region of liver in STZ-diabetic rat transplanted with stage 4 islet-like cell clusters. The cell size and morphology of these human insulin-positive staining cells are similar with hepatocytes. The human origin of the engrafted cells was confirmed by immunohistochemistry using the anti-human nuclei monoclonal antibody (Fig. 7B-c, in green). The merged image of human insulin and human nuclei immunostaining was shown in Figure 7B-d. There was no human insulin and human nuclei immunostaining in the sham liver of STZ-diabetic rat without transplantation of cell clusters (Fig. 7B-h-j). These findings indicated that implanted islet-like cell clusters and human insulin and nuclei-positive staining liver cells are simultaneously existed in the liver after transplantation of stage 4 islet-like cell clusters for 3 months. Next, we further investigated these human insulin and nuclei-positive staining liver cells using electron microscopy (Figs. 7B-e-g). These cells possessed secretory granules densely packed in the cytoplasm. These secretory granules were small (300–600 nm in diameter) and possessed electron-dense cores. Crystalline formation was detected in the cores of the granules, a feature that is characteristic of secretory granules in normal pancreatic cells of rodents.

## Discussion

Recent studies have demonstrated the feasibility of generating insulin-producing cells obtained from progenitor cells of various cellular sources [8]–[15]. Despite their promising potential, it also proved difficult to obtain enough autologous adult stem cells from these organs. Some obstacles, such as the immune rejection and autoimmunity against newly formed β-cells derived from pancreatic stem cells, still remain. To overcome these limitations, we explored the possibility of using HUMSCs in Wharton's Jelly of the umbilical cord as a source for cellular differentiation into insulin-producing cell clusters under de novo culture conditions. HUMSCs in Wharton's Jelly of the umbilical cord are easily obtained compared with embryonic and other stem cells. In our previous and present studies, approximately 1×10^6^ HUMSCs were collected from a 20-cm of umbilical cord [21]. The doubling time of HUMSCs in 10% FBS-DMEM is 3 days. Wharton's Jelly cells have been cultured for more than 80 population doublings with no indications of senescence, changes in morphology, increased growth rate, or change in ability to differentiate into neurons [20]. The transformed HUMSCs in the striatum have been demonstrated to be viable 4 months after transplantation without the need for immunological suppression [21]. The study of Weiss and colleagues has also shown the stem cells from human umbilical cord Wharton's Jelly that were transplanted into normal rats did not produce brain tumors, rotational behavior, or a frank host immune rejection response [22]. In the present study, HUMSCs were induced to transform into pancreatic islet-like cell clusters *in vitro* through stepwise culturing in neuron-conditioned medium and stem cell-conditioned medium. These islet-like cell clusters were shown to contain human C-peptide and release insulin in response to physiological glucose levels. Real-time RT-PCR analysis also showed the enhancement of insulin and other pancreatic β-cell-related genes, such as *Pdx1*, *Hlxb9*, *Nkx2.2*, *Nkx6.1*, and *Glut-2* in these islet-like cell clusters. Moreover, the hyperglycemia was significantly under control in streptozotocin-induced diabetic rats after xenotransplantation of these human pancreatic islet-like cell clusters. Even without the use of immunosuppressants, there was no evidence of rejection. Therefore, HUMSCs in Wharton's Jelly of the umbilical cord represent not only a safe and abundant source for large quantities of stem cells, but also one that is easily manipulated and free of ethical problems.

Pancreatic endocrine cells were originally proposed to be of neuronal origin, because of its neuronal-marker expression. On the other hand, it has been unequivocally established that they arise from the endoderm [23]. In some invertebrate species, such as *Drosophila,* brain neurons were the main source of circulating insulin [24], [25]. Insulin gene transcription has also been identified in the vertebrate brain [26]. It has been shown that multipotent precursor cells from the adult mouse pancreas can form clonal colonies, which express both neural and pancreatic precursor markers, generating distinct populations of neurons and glial cells, pancreatic endocrine cells, and pancreatic exocrine and stellate cells [27]. These findings indicated the similarities between islet cells and neurons. Treutelaar and colleagues have reported that nestin-lineage cells contribute to the microvasculature but not endocrine cells of the islet [28]. Delacour and colleagues have also shown that nestin is expressed in the pancreatic exocrine cell lineage, but the consistent nestin expression is not a major feature of islet endocrine progenitor cells [29]. However, recent study of Hori and colleagues suggested that brain-derived human neural progenitor cells, when exposed to a series of signals that regulate in vivo pancreatic islet development, form clusters of glucose-responsive insulin-producing cells [13]. In the present study, the neuronal conditioned medium was made from rat brain, on which HUMSCs grew and differentiated into nestin-positive cells. Expression of nestin reflects the differentiating state of neural progenitor cells in the developing central nervous system [30], [31]. These nestin-positive cells were then changed into insulin-producing cells under the medium containing high glucose, high insulin, nicotinamide, and B27, later forming the islet-like cell clusters. Nicotinamide is a poly (ADP-ribose) synthetase inhibitor known to differentiate and increase β-cell mass in cultured human fetal pancreatic cells [32], as well as to protect β-cells from desensitization induced by prolonged exposure to large amounts of glucose [33]. Our immunocytochemical analysis showed that stage 4 islet-like cell clusters do not express nestin, implying that these cell clusters are non-neuronal. The real-time RT-PCR analysis showed the enhancement of insulin and other pancreatic β-cell-related genes also imply that these insulin-producing cells in islet-like cell clusters are β-cells or β-cell-like cells.

High glucose condition has been useful for isolating and maintaining progenitor cell populations from the nervous systems [34], [35] and for permitting differentiation by neurogenic cells [13], [36]. Hori and colleagues have found that human neural progenitors could aggregate in high glucose condition to produce a cell cluster [13]. In our study, the nestin-positive cells developed into insulin-producing cells under high glucose condition. However, prolonged exposure to high glucose condition not only severely reduced insulin expression in β-cells, but also resulted in β-cell apoptosis [37], [38]. Unexpectedly, we found that supplemented stem cell-conditioned medium provides the functions of protection and regeneration in stage 4 islet-like cell clusters. These findings imply that some factors in stem cell-conditioned medium promote islet-like cell clusters differentiation and prevent glucose-induced toxicity. The mechanism of action of stem cell-conditioned medium on islet-like cell clusters needs to clarify in the future.

Type 1 diabetes has always been known as a disease of childhood, but more recent epidemiological studies have indicated that the incidence is comparable in adults [39]. Type 1 diabetes has a strong genetic component, inherited mainly through the HLA complex, although the factors that trigger onset of clinical disease remain unknown [1], [39]. Susceptibility for type 1 diabetes is largely inherited by genes predominantly in the HLA genotypes DR and DQ. Both high risk and protective HLA haplotypes have been identified [1]. These susceptibility genes are thought to be important regulators of the cellular immune response. Moreover, HUMSCs in Wharton's Jelly of the umbilical cord have been demonstrated to have genetic and surface markers of mesenchymal stem cells: positive for CD10, CD13, CD29, CD44, and CD90 and negative for CD14, CD33, CD56, CD31, CD34, CD45, and HLA-DR [22]. In the present study, therefore, lack of HLA-DR maybe the one of reasons that differentiated HUMSCs can survive during xenotransplantation, and implies that the differentiated HUMSCs can appear invisible to the effects of the underlying autoimmunity.

Cell fusion has recently been suggested to possess the ability to produce viable cells and to induce cells from one tissue type to form other tissue types after transplantation [40]–[43]. It has been demonstrated that transplanted bone-marrow-derived cells fuse *in vivo* with hepatocytes in liver, Purkinje neurons in the brain, and cardiomyocytes in the heart [42]. Wang and colleagues have suggested that hepatocytes derived form bone marrow arise from cell fusion and not by differentiation of haematopoietic stem cells [43]. In the present study, from the analysis of immunohistochemistry, the liver transplanted with cell clusters not only contained islet-like cell clusters, but also possessed human insulin and nuclei-positive staining liver cells. The detection by electron microscopy showed that these human insulin and nuclei-positive staining liver cells contained one nucleus, a mitochondria-rich cytoplasm, and a very well developed endoplasmic reticulum; but there were multiple dense secretory granules and less glycogen storage granules in the cytoplasm. Therefore, cell fusion of transplanted cells into liver cells is suspected. Since transplanted cell clusters could still be found 3 months after transplantation, cell fusion is thought to be one of the reason that no rejection occurred in the animals, which implanted the stage 4 islet-like cell clusters. Moreover, our results showed that high blood glucose level of the STZ-induced diabetic rats was rapidly declined to less than 200 mg/dl on day 3 after transplantation of stage 4 cell clusters. If there were not enough functional islet-like cell clusters in the early stage of transplantation, the blood glucose level should not be normalized so rapidly. Nevertheless, the maintenance of normalized blood glucose level during transplantation may need the formation of fused liver cells (human insulin and nuclei-positive staining and contained secretory granules) through a possible cell fusion pathway. The detail mechanism needs further investigation in the future.

In conclusion, in this study, we successfully differentiate HUMSCs into mature islet-like cell clusters, that possess insulin-producing ability *in vitro* and *in vivo*. HUMSCs in Wharton's Jelly of the umbilical cord seem to be a favorable source of stem cells for conversion into insulin-producing cells, because of its large potential donor pool, rapid availability, absence of discomfort to the donor, and low risk of rejection. Therefore, HUMSCs in Wharton's Jelly of the umbilical cord have the potential to become an excellent candidate in β-cell replacement therapy of diabetes.

## Materials and Methods

### Preparation of Human Umbilical Mesenchymal Stem Cells (HUMSCs)

With the consent of the parents, fresh human umbilical cords were obtained after birth and collected in HBSS (Gibco) at 4°C. Following disinfection in 75% ethanol for 30 s, the umbilical cord vessels were cleared off in HBSS. HUMSCs were prepared as previously described [21]. The mesenchymal tissue (in Wharton's jelly) was diced into cubes of about 0.5 cm^3^ and centrifuged at 250×g for 5 min. Following removal of the supernatant fraction, the precipitate (mesenchymal tissue) was washed with serum-free Dulbecco's modified Eagle's medium (DMEM) (Gibco) and centrifuged at 250×g for 5 min. The mesenchymal tissue was treated with collagenase at 37°C for 18 h, washed and further digested with 2.5% trypsin (Gibco) at 37°C for 30 min. Fetal bovine serum (FBS) (Hyclone) was added to the mesenchymal tissue to neutralize the excess trypsin. The dissociated mesenchymal cells were further dispersed by treatment with 10% FBS-DMEM and counted. The mesenchymal cells were then used directly for cultures or stored in liquid nitrogen for later use. Moreover, in some experiments, the stem cell-conditioned medium (SCM) was used. The preparation of SCM: the mesenchymal cells were suspended in 12 ml of DMEM/F12, 2% FBS, 1 mM glutamine, 10 mM nicotinamide. After 3 days culture, the medium (SCM) was ready for usage in stage 4 of HUMSCs differentiation.

### Preparation of Neuronal Conditioned Medium (NCM)

NCM was prepared as previously described [21]. Seven-day postnatal Sprague-Dawley rats were anesthetized by intraperitoneal injection of 10% chloride hydrate. The brain was removed, placed in Ca^2+^/Mg^2+^-free buffer (Gibco) and centrifuged at 900 rpm for 5 min. Following removal of the supernatant fraction, 10% FBS-DMEM was added to the precipitate (brain tissue). The brain tissue suspension was triturated 15 times for dispersal into single cells. The cells were suspended in 10% FBS-DMEM and incubated at 37°C in 5% CO_2_ and 95% O_2_. The next day, AraC (Sigma-Aldrich) at a concentration of 2 µM was added. On the 5^th^ day of culture, the culture medium (NCM) was removed to be used for the culture of umbilical mesenchymal cells. To generate nestin-positive cells, HUMSCs were cultured in NCM alone, which was replaced every other day.

### Generation of Islet-Like Cell Clusters from Undifferentiated HUMSCs in Vitro

In vitro differentiation of HUMSCs into islet-like cell clusters was carried out by four stages. In stage 1, undifferentiated HUMSCs were cultured and expanded in 10% FBS/DMEM for 3 to 6 days. In stage 2, HUMSCs were cultured in NCM alone for 7 days, which was replaced every other day to induce nestin-positive cells. In stage 3, cells were cultured in 2% FBS/DMEM (25 mM glucose)/F12 medium and supplemented with 10 mM nicotinamide and B27 (GIBCO) for 7 days. In stage 4, differentiated premature islet-like cell clusters were cultured in stage 3 medium and supplemented with SCM for 14 days. The outcome of this procedure produced many islet-like cell clusters, which could express insulin and glucagon as shown in figures 1 and 2.

### Human Insulin and C-Peptide Quantification and In Vitro Insulin Secretion Assay

For in vitro insulin secretion assay, twenty stage 4 cell clusters were handpicked, washed with PBS 5 times to prevent insulin assay cross reaction with medium, and then cultured in 2% FBS/DMEM (5.5 mM glucose) without B27 and nicotinamide for 24 h. Next, clusters were washed with PBS 5 times again and cultured in 2% FBS/DMEM (25 mM glucose) without B27 and nicotinamide for 24 h. Supernatants from medium for 5.5 or 25 mM glucose-stimulated clusters were harvested for enzyme-linked immunosorbent assay (ELISA)-based quantification of released insulin (Mercodia, Sweden). Moreover, C-peptide in medium of stage 3 and 4 cell clusters was determined using ELISA kit (Mercodia, Sweden), according to the manufacturer's instruction.

### RNA Extraction and Real-Time Polymerase Chain Reaction

Total RNA was extracted from stage1, stage3, stage4, human islets and RIN-m5F cells using TRIzol (Gibco, California USA) and following the manufacturer's recommendations. The resulting products were amplified using the following primers: insulin (192 bp), forward 5′-TTCTTCTACACACCCAAGAC-3′, reverse 5′-CTAGTTGCAGTAGTTCTCCA-3′, Pdx1 (198 bp), forward 5′-TGGATGA- AGTCTACCAAAGC-3′, reverse 5′-GGTCAAGTTCAACATGACA- G-3′, Hlxb9 (197 bp), forward 5′-TAAGATGCCCGACTTCAAC-3′, reverse 5′-TGGAA- CCAAATCTTCACCT-3′, Nkx2.2 (227 bp), forward 5′-ATGTAAACGTT- CTGACAACT-3′, reverse 5′-TTCCATATTTGAGAAATGTTTGC-3′, Nkx6.1 (215 bp), forward 5′-CTATTCGTTGGGGATGACA-3′, reverse 5′-AGCTGC- GTGATTTTC- T-3′, Glut-2 (196 bp), forward 5′-GAATAACATTGCCAAACGTC-3′, reverse 5′-AAGAAC- CATCAGCATGTC-3′. Rat insulin primer, forward 5′-TGG TTT CTT CTA CAC ACC C-3′, reverse 5′-CTC AGT TGC AGT AGT TCT C-3′. cDNA was prepared from 5 µg of total RNA following DNase treatment and 10 ng RNA equivalent used for PCR with specific primers in the presence of SYBR Green I (Light Cycler™-FastStart DNA Master SYBR green I (Rouche)), as previously described [44]. A melt curve analysis was performed at the end of each reaction. Expression levels were normalized to individual GAPDH (internal control). The profile was obtained by plotting relative gene expression levels comparing to undifferentiated HUMSCs (stage 1 cells).

### RIN-m5F cells and human islets culture

RIN-m5F cells (ATCC, CRL-11605) were cultured in RPMI 1640 medium with 2 mM L-glutamine adjusted to contain 1.5 g/L sodium bicarbonate, 4.5 g/L glucose, 10 mM HEPES, and 1 mM sodium pyruvate, and supplemented with 10% fetal bovine serum and 100 µg/ml streptomycin, 100 units/ml penicillin at 37°C in an atmosphere of 95% air/5% CO_2_. Moreover, human islets were isolated by collagenase digestion from the surrounding non-tumor pancreatic tissue, which was taken from patient with benign pancreatic tumor after obtaining written informed consent. This experiment was approved by the Research Ethics Committee at the National Taiwan University Hospital. Human islets were cultured in CMRL 1066 medium (5.5 mM glucose) containing 10% FCS, 100 µg/ml streptomycin, 100 units/ml penicillin, 1 mM sodium pyruvate, 2 mM L-glutamine, and 2 mM HEPES.

### Insulin Secreting Cells Transplantation

Sprague-Dawley rats (200–250 g) were purchased from the Animal Center of the College of Medicine, National Taiwan University (Taipei, Taiwan). The Animal Research Committee of College of Medicine, National Taiwan University, conducted the study in accordance with the guideline for the care and use of laboratory animals. Experimental diabetes was induced 7 days before transplantation by intraperitoneal injection of streptozotocin (STZ, Sigma) 50 mg/kg/day for 2 days. Draw blood from tail vein was collected to detect blood glucose levels with a SURESTEP blood glucose meter (Lifescan) before STZ injection, every week after STZ injection, and once additionally three days after transplantation. One week after STZ injection the blood glucose levels were over 400 mg/dl. Under anesthetized by intramuscular injection of 10% chloride hydrate, the stage 4 insulin secreting islet-like cell clusters (about 2×10^6^ cells) were transplanted into the liver of the STZ-induced diabetic rats via laparotomy with a 22^#^ needle slowly injected into liver parenchyma. Another group of STZ-DM animals were treated with undifferentiated HUMSCs (2×10^6^), and the other sham animals were treated with normal saline. In some experiments, intraperitoneal glucose tolerance (IPGT) test was performed with 1g/kg of glucose.

### Immunocytochemistry

Cell clusters were fixed with 4% paraformaldehyde in 0.1 M phosphate buffer for 20 min and then washed with 0.1 M phosphate buffer. They were then treated with a blocking solution for 30 min to prevent nonspecific antibody-antigen binding. The cells clusters were then reacted with primary antibodies: mouse anti-nestin, 1∶100 (Abcam); mouse anti-human insulin 1∶30 (Santa Cruz Biotechnology); mouse anti-human nuclei, 1∶30 (Chemicon); rabbit anti-human glucagon 1∶50 (Serotec), at 4°C for 18 h, washed with 0.1 M phosphate-buffered saline (PBS), reacted with secondary antibodies: rhodamine-conjugated goat anti-mouse IgG, 1∶50 for nestin and insulin; rhodamine-conjugated goat anti-rabbit IgG, 1∶50 for glucagons at room temperature for 1 h. The cell clusters were then observed under a fluorescence microscope. In some experiments, after reacted with primary antibody (mouse anti-human insulin) and secondary antibody, the cell clusters reacted with ABC complex (ABC KIT, Vector laboratories) at room temperature for 1 h, washed with 0.1 M PBS, and finally developed with 3,3′-diaminobenzidine (DAB) (1 mM DAB, 50 mM Tris buffer, pH 7.6, and 0.015% H_2_O_2_).

### Immunohistochemistry

Livers were isolated and fixed in 10% neutral-buffered formalin. After infiltrating with 30% sucrose solution in PBS, cut the tissue using frozen sections and paraffin sections method. For antigen retrieval, tissue sections on slides were immersed in borate buffer solution (pH 8) jar and placed in pressure oven for about 20 min until the cooker reached its maximum pressure. It was then heated for another 5 min at maximum pressure. Thereafter, the pressure was reduced and cooled in a bath of tap water. Then the sections incubate in blocking solution at room temperature for 6 hours. And they were incubated overnight at 4°C with the following primary antibodies and dilutions: mouse anti-human nuclei monoclonal antibody (Chemicon, 1∶100); guinea pig anti-human insulin antibody (Linco Research, 1∶100). These two primary antibodies were specific for human. Wash the slides three times for 10 min in 0.1 M PBS, then incubated with secondary antibodies (diluted according to the manufacturer's instructions) for 1 h in 0.1 M PBS at room temperature. Secondary antibodies: FITC-conjugated goat anti-mouse IgG, 1∶50 for human insulin or rhodamine-conjugated goat anti-rabbit IgG, 1∶50 for human nuclei in liver sections (Chemicon). Cell nuclei were visualized by incubating 5 min with 4′-6 ′diamindino-2-phenylindole (DAPI) (Sigma, 1∶2000) in 0.1 M PBS. The immunohistochemistry identification of human cells was performed by using a primary mouse anti-human nuclei monoclonal antibody (Chemicon), specific for all human cell types.

### Electron Microscopic Study for Transplanted Liver

The rats with stage 4 cell clusters transplantation were sacrificed. The livers were isolated and fixed with 4% paraformaldehyde and 2.5% glutaraldehyde, and then the samples were embedded in epoxy resin (Spurr; EMS). Ultra-thin sections were cut in an ultramicrotome, stained with 2% uranyl acetate for 15 min and 3% lead citrate for 3 min. Then the sections were observed under a transmission electron microscope.

### Statistical Analysis

The data are given as means±SEM. The significance of difference was evaluated by the paired Students' t-test. When more than one group was compared with one control, significance was evaluated according to one-way analysis of variance (ANOVA). Statistical difference was indicated by P<0.05.
